# Clinical outcomes of serum potassium in patients with percutaneous coronary intervention: insights from a large single-center registry

**DOI:** 10.3389/fcvm.2023.1216422

**Published:** 2023-08-10

**Authors:** Bingbing Ke, Aidong Shen, Hui Qiu, Weiping Li, Hui Chen, Hongwei Li

**Affiliations:** ^1^ Department of Cardiology, Cardiovascular Center, Beijing Friendship Hospital, Capital Medical University, Beijing, China; ^2^ Beijing Key Laboratory of Metabolic Disorder Related Cardiovascular Disease, Beijing, China

**Keywords:** serum potassium, MACCE, percutaneous coronary intervention, retrospective study, cohort

## Abstract

**Background:**

Serum potassium homeostasis plays an important role in myocardial electrical stability, but the impact of altered serum potassium levels on the major adverse cardiovascular and cerebral events (MACCE) in patients with percutaneous coronary intervention (PCI) has not been evaluated.

**Aim:**

To evaluate the association between serum potassium level and the risk of MACCE in PCI patients.

**Materials and methods:**

This retrospective study involved 8,263 in-patients from a single-center registry who were successfully treated with PCI between January 2003 and December 2020. Clinical data were collected for 24 h after admission. Data were analyzed from June 2003 to December 2021. The primary outcome was MACCE, defined as a composite of all-cause death, myocardial infarction, revascularization, stroke, and heart failure-related hospitalization.

**Results:**

The median [interquartile range (IQR)] follow-up for all patients was 4.0 (2.1, 5.8) years, and 1,632 patients (19.7%) were diagnosed with MACCE. High serum potassium levels were associated with a 20% increased risk of MACCE (hazard ratio [HR]: 1.20, 95% confidence interval [CI]: 1.05–1.38, *P *= 0.008) and 72% increased risk of all-cause death (HR: 1.72, 95% CI: 1.39–2.14, *P *< 0.001). Multivariate Cox analysis showed that the risk of MACCE was higher in patients at the highest quartile of serum potassium (Q4 vs. Q1: adjusted HR: 1.18, 95% CI: 1.02–1.35, *P* = 0.026). Moreover, a higher serum potassium level was always associated with a higher risk of all-cause death (Q4 vs. Q1: adjusted HR: 1.50, 95% CI: 1.17–1.91, *P* = 0.001). A U-shaped relationship between serum potassium levels, MACCE, and all-cause death was derived in patients undergoing PCI. Serum potassium levels, maintained within the range of 3.8–4.0 mmol/L before PCI, exhibited the lowest risk of associated MACCE and all-cause death.

**Conclusion:**

Our results demonstrate that the serum potassium level could be associated with higher risks of MACCE and all-cause death in PCI patients. In particular, serum potassium levels maintained at 3.8–4.0 mmol/L before PCI could lower the risk of MACCE and all-cause death.

## Introduction

Physiological potassium level is essential for mediating normal heart functions through the stabilization of cardiac electrical conduction. Conditions like hypo- and hyperkalemia are caused due to electrolyte imbalance and may be closely related to malignant arrhythmia and sudden death (1–3). It's suggested that hypokalemia can be associated with ventricular arrhythmias in the setting of acute coronary syndrome (ACS) (4). Consistently, recent studies have confirmed the prognostic relevance of higher serum potassium levels in ACS patient populations (5).

Current guidelines of ACS management recommend that the target potassium level should be at least 4.0 mmol/L (6). The evidence behind this recommendation has been established by the clinical practice of repletion of potassium to reduce the risk of ventricular arrhythmias. However, in a large retrospective cohort study using the CERNER Health Facts database, Goyal et al. have shown that the rate of in-hospital mortality is the lowest in patients with potassium levels ranging from 3.5 to 4.0 mmol/L (7). Notably, the study challenged prior recommendations of a target serum potassium level of greater than 4.0 mmol/L. Despite this evidence, the optimal serum potassium target remains a debatable issue in the setting of ACS. In the treatment of coronary heart disease (CHD), the efficacy of the percutaneous coronary intervention (PCI) using cardiac catheterization technology to open the narrowed or occluded coronary artery lumen has been well-established and widely used to improve myocardial blood perfusion. Moreover, the effect of serum potassium imbalance on patients with PCI has never been explored in detail.

Based on the above indirect evidence, in clinical practice, PCI patients with potassium levels lower than 4.0 mmol/L are initially treated with potassium supplementation. Therefore, whether serum potassium concentrations have similar clinical implications in patients with PCI needs further investigation. Presently, there is no solid evidence confirming the pathological role of altered potassium levels in inducing major adverse cardiovascular and cerebral events (MACCE) in PCI patients. The optimal range of serum potassium levels in these patients is still a matter of clinical concern. To address this unresolved question, we assessed whether altered serum potassium levels were associated with an increased risk of MACCE in PCI patients and also sought to establish an optimal range of serum potassium levels in this subset of patients.

## Materials and methods

### Study population and study cohort

**Figure 1:**
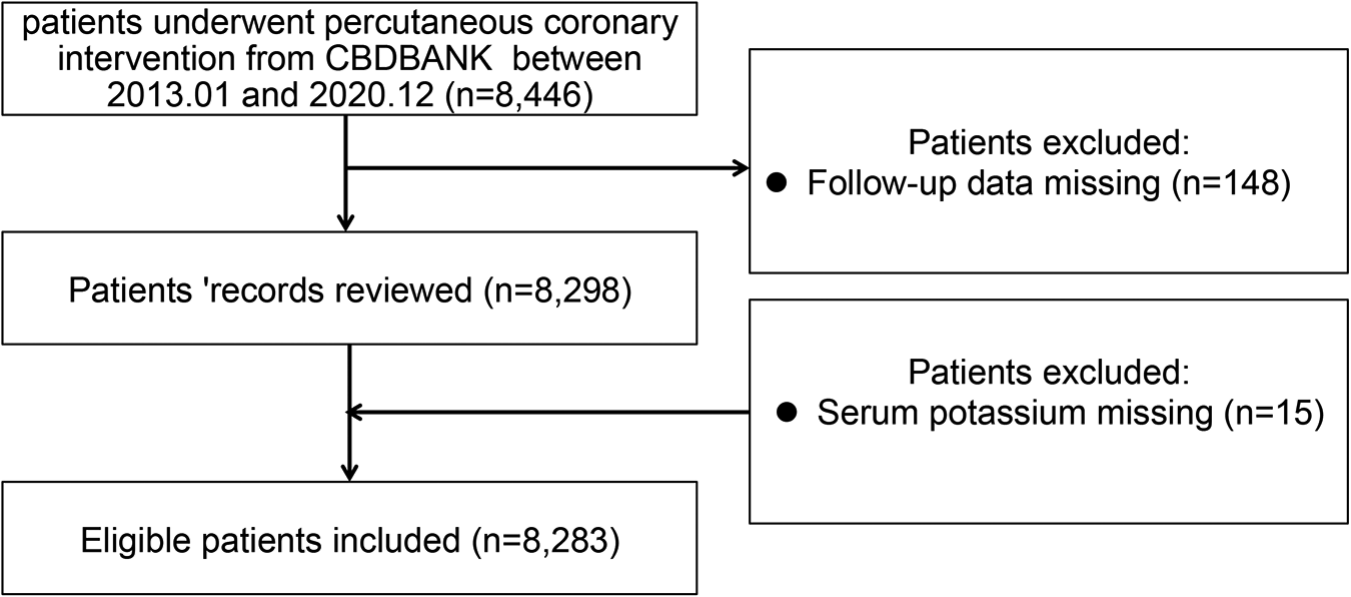
Flowchart of the patient selection process. CBDBANK: Cardiovascular Center Beijing Friendship Hospital Database Bank.

Study subjects were enrolled in the registration study of PCI in Beijing Friendship Hospital from January 2003 to December 2020. All enrolled participants were in-patients with available medical and clinical records of 24 h post-admission. The patient's demographic, clinical, procedural, and angiographic data were subsequently curated in the Cardiovascular Center Beijing Friendship Hospital Data Bank (CBDBANK). Briefly, adult patients (≥18 years) who underwent a successful PCI [defined by a final Thrombolysis in Myocardial Infarction (TIMI) with a flow grade of 3 and <10% of residual stenosis by visual estimation] in at least one epicardial coronary artery were eligible for inclusion. Exclusion criteria included patients who failed PCI treatment and had a known inability to follow instructions or comply with follow-up procedures. The research protocol was approved by the Medical Ethics Committee of Beijing Friendship Hospital in compliance with the Declaration of Helsinki. Written informed consent was obtained from all participating patients. Figure 1 describes the patient selection process. The first participant was recruited in June 2003, and the follow-up examinations were terminated in December 2021 (NCT05728580).

### In-patient serum potassium measurements and outcomes

The admission serum potassium level was defined as the first venous serum potassium level obtained at admission to the hospital. Information on adverse events was obtained from the CBDBANK. Patients who met the inclusion and exclusion criteria were then routinely followed up at 1 month, 3 months, 6 months, and then once every year after discharge, either via telephone or outpatient service. The primary outcome was a composite of MACCE (defined as a composite of all-cause death, myocardial infarction (MI), revascularization, stroke, and heart failure (HF)-related hospitalization). Definitions of events are in accordance with those proposed by the Standardized Data Collection for Cardiovascular Trials Initiative (8). All-cause death incidences included both cardiac and non-cardiac deaths. MI was defined as the detection of a rise and/or fall of cardiac biomarker values (preferably cTn) with at least 1 value of >99th percentile of the URL and at least 1 of the following parameters: symptoms of myocardial ischemia; new or presumably new onset of significant ST-segment-T wave (ST-T) changes or new left bundle branch blocks on the ECG; development of pathological Q waves on the ECG; imaging evidence of recent loss of viable myocardium or regional wall motion abnormality, and identification of an intracoronary thrombus by angiography. Revascularization was defined as unplanned readmission for stent or bypass grafting treatment. Stroke was defined as a recent onset of neurologic deficits originating from vascular brain lesions and lasting more than 24 h. HF-associated hospitalization was defined as an event in which the patient was admitted to the hospital with a primary diagnosis of HF, the length of stay was at least 24 h (or extends over a calendar date), exhibited recent or worsening symptoms of HF on presentation, and received initiation or intensification of HF-specific treatments.

### Statistical analysis

The sample size calculation was based on estimates for mean 5-year follow-up rates for the composite primary endpoint of MACCE. The Nordic–Baltic–British Left Main Revascularisation (NOBLE) study reported that the rate of MACCE was 28% at 5 years (9). We set the alpha risk to 0.05 and the power to 80%. At least 1,304 patients were needed to detect a difference of 5% in MACCE at 5 years.

Continuous variables were expressed as mean ± standard deviation (SD) or median (interquartile range, IQR) and compared using Student's *t*-test or Wilcoxon Rank Sum test, as appropriate. Categorical variables were presented as counts and percentages and compared using the chi-squared (*χ*^2^) test or Fisher's exact test, as appropriate. Kaplan-Meier survival curves and the log-rank test were applied to assess the difference between the curves. A multivariate Cox Proportional-Hazards model was used to determine the hazard ratio (HR) and the corresponding 95% confidence interval (CI). Model 1 was adjusted by age and gender. Model 2 was adjusted by: model 1 plus diabetes mellitus (DM), hypertension, the prior occurrence of MI and stroke, body mass index (BMI); discharge medications, like aspirin, clopidogrel, ticagrelor, *β*-blockers, angiotensin-converting enzyme (ACE) inhibitor/angiotensin receptor blocker (ARB), statins, diuretics and routine blood works including total cholesterol (TC), total triglyceride (TG), high-density lipoprotein cholesterol (HDL-C), low-density lipoprotein cholesterol (LDL-C) and estimated glomerular filtration rate (eGFR). To test the model discrimination, Harrell's C-index was performed. A multivariate Cox model with restricted cubic splines (RCS) was built to evaluate the linearity assumption of the association of serum potassium level with MACCE and all-cause death. Stratification analysis was conducted to determine whether the association of serum potassium level with MACCE and all-cause death could be altered by a non/ST-elevation myocardial infarction (NSTEMI or STEMI), and unstable/stable angina pectoris (UAP or SAP). Due to missing values (<5% for all covariates), multivariate imputation by chained equations was used that considered 10 multiple imputations. All tests were two-tailed with a Type 1 error rate of 0.05. All analyses were performed using IBM SPSS version 23 (SPSS Inc., Illinois, USA), and R version 3.6.3 (R Foundation for Statistical Computing, Vienna, Austria).

## Results

### Baseline characteristics

The study flow chart is shown in Figure 1. Between January 2003 and December 2020, a total of 8,446 patients with PCI were consecutively recruited for this study. Then, eight patients were excluded due to their missing information on serum potassium levels, and 148 patients were excluded due to no follow-up data. Finally, 8,283 patients were included in the analysis. The median serum potassium level was 4.05 (3.84–4.29) mmol/L with a range of 2.63–7.35 mmol/L, and the prevalence of normokalemia (3.5–5.5 mmol/L) was 95.2%. The median age of the subjects was 63 years, and 71.2% of them were male. In Table 1, the association of baseline characteristics across different serum potassium quartiles is presented. We found that serum potassium quartiles were not significantly associated with either hyperlipidemia or a prior history of MI/stroke. Higher serum potassium levels were detected mostly in elderly female patients, while patients with higher serum potassium quartiles exhibited a higher proportion of DM, hypertension, and chronic renal failure symptoms. Moreover, this subset of patients also presented an abnormal lipid profile, including higher levels of TC, TG, HDL-C, LDL-C, and creatinine (Cr). However, a lower serum potassium (but not sodium) level was associated with higher BMI, systolic/diastolic blood pressure (S/D BP), eGFR, and left ventricular ejection fraction (LVEF). Although lower serum potassium levels were associated with increased use of ticagrelor and calcium channel antagonists, patients with higher serum potassium levels received more medications such as clopidogrel and diuretics. However, the latter group did not require more of other discharge medications, including aspirin, β-blockers, ACEI/ARBs, nitrates, or statins. Moreover, higher serum potassium levels were associated with an overall higher prevalence of left main disease. There was no difference in diseased vessels or stent numbers between higher and lower potassium levels.

**Table 1:** Baseline characteristics according to potassium quartiles.

Variable	Potassium (mmol/L) (*n* = 8,283)				*P*-value
	Quartile 1	Quartile 2	Quartile 3	Quartile 4	
	(2.63–3.84, *n* = 2,131)	(3.84–4.05, *n* = 2,025)	(4.05–4.29, *n* = 2,117)	(4.29–7.35, *n* = 2,010)	
Clinical characteristics					
Age (years)	62 (56,69)	63 (56,70)	63 (57,72)	65 (58,74)	<0.001
Female, *n* (%)	561 (26.3)	549 (27.1)	606 (28.6)	673 (33.5)	<0.001
Body mass index (kg/m^2^)	25.83 (23.66,28.23)	25.71 (23.59,27.78)	25.47 (23.51,27.68)	25.40 (23.31,27.68)	<0.001
SBP (mmHg)	130 (119,143)	130 (119,140)	129 (118,140)	130 (118,141)	0.001
DBP (mmHg)	76 (70,83)	75 (68,82)	74 (68,80)	75 (68,81)	<0.001
HR (b.p.m.)	71 (63,79)	70 (63,78)	70 (63,79)	70 (64,80)	0.263
Diabetes mellitus, *n* (%)	802 (37.6)	735 (36.3)	727 (34.3)	783 (39.0)	0.016
Hypertension, *n* (%)	1,494 (70.1)	1,365 (67.5)	1,396 (65.9)	1,374 (68.4)	0.031
Hyperlipidaemia, *n* (%)	984 (46.2)	957 (47.3)	1,015 (47.9)	905 (45.0)	0.256
Myocardial infarction, *n* (%)	174 (8.2)	180 (8.9)	185 (8.7)	197 (9.8)	0.32
Stroke, *n* (%)	329 (15.4)	315 (15.6)	303 (14.3)	298 (14.8)	0.651
Chronic renal failure, *n* (%)	51 (2.4)	46 (2.3)	51 (2.4)	118 (5.9)	<0.001
Laboratory characteristics					
Random glucose (mg/dl), median (IQR)	5.78 (4.97,7.31)	5.67 (4.93,7.12)	5.63 (4.94,7.08)	5.76 (4.98,7.39)	0.049
Sodium (mmol/L)	140.7 (138.9,142.4)	140.7 (139.0,142.4)	141 (139,142.6)	140.8 (138.7,142.6)	0.199
Creatinine (mg/dl), median (IQR)	75.3 (64.5,86.1)	76.3 (66.7,88.3)	79.1 (68.9,90.1)	82.95 (71.6,97.8)	<0.001
eGFR (ml/min/1.73 m^2^)	90.78 (78.20,103.56)	88.51 (74.88,101.46)	84.82 (71.29,98.49)	78.98 (62.88,92.53)	<0.001
TC (mg/dl), median (IQR)	4.27 (3.60,4.94)	4.31 (3.65,5.03)	4.35 (3.68,5.04)	4.35 (3.64,5.08)	0.031
TG (mg/dl), median (IQR)	1.51 (1.08,2.14)	1.49 (1.08,2.10)	1.47 (1.08,2.11)	1.43 (1.04,2.06)	0.033
HDL-C (mg/dl), median (IQR)	0.98 (0.85,1.14)	0.99 (0.85,1.16)	1.01 (0.88,1.18)	1.03 (0.88,1.22)	<0.001
LDL-C (mg/dl), median (IQR)	2.43 (1.95,2.92)	2.47 (1.97,2.97)	2.49 (2.01,2.98)	2.49 (2.00,3.02)	0.03
LVEF (%)	65 (59,69)	65 (59,69)	65 (59,69)	64 (57,69)	<0.001
Medications at discharge					
Aspirin, *n* (%)	2,108 (98.9)	1,998 (98.7)	2,095 (99.0)	1,987 (98.9)	0.82
Clopidogrel, *n* (%)	1,633 (76.6)	1,637 (80.8)	1,740 (82.2)	1,664 (82.8)	<0.001
Ticagrelor, *n* (%)	407 (19.1)	316 (15.6)	306 (14.5)	267 (13.3)	<0.001
β-blockers, *n* (%)	1,699 (79.7)	1,629 (80.4)	1,709 (80.7)	1,626 (80.9)	0.786
ACEI/ARBs, *n* (%)	1,360 (63.8)	1,298 (64.1)	1,318 (62.3)	1,277 (63.5)	0.617
Calcium channel antagonist, *n* (%)	765 (35.9)	667 (32.9)	647 (30.6)	594 (29.6)	<0.001
Nitrates, *n* (%)	1,815 (85.2)	1,712 (84.5)	1,797 (84.9)	1,697 (84.4)	0.908
Statins, *n* (%)	2,105 (98.8)	1,989 (98.2)	2,098 (99.1)	1,983 (98.7)	0.094
Diuretics, *n* (%)	183 (8.6)	117 (5.8)	149 (7.0)	206 (10.2)	<0.001
PCI characteristics					
Diseased vessels, *n* (%)					0.248
1-vessel	179 (8.4)	185 (9.1)	176 (8.3)	145 (7.2)	
2-vessel	353 (16.6)	320 (15.8)	314 (14.8)	332 (16.5)	
3-vessel	1,599 (75.0)	1,520 (75.1)	1,627 (76.9)	1,533 (76.3)	
Left main disease, *n* (%)	206 (9.7)	240 (11.9)	258 (12.2)	278 (13.8)	0.001
CAD					0.064
STEMI	507 (23.8)	452 (22.3)	479 (22.6)	525 (26.1)	
NSTEMI	397 (18.6)	387 (19.1)	401 (18.9)	331 (16.5)	
UAP	1,071 (50.3)	1,027 (50.7)	1,094 (51.7)	1,023 (50.9)	
SAP	156 (7.3)	159 (7.9)	143 (6.8)	131 (6.5)	
Status of procedure					0.078
Selective	1,760 (82.6)	1,703 (84.1)	1,804 (85.2)	1,665 (82.8)	
Emergent	371 (17.4)	322 (15.9)	313 (14.8)	345(17.2)	
Drug-eluting stent	2,071(97.2)	1,963(96.9)	2,064(97.5)	1,965(97.8)	0.385
Stent number, median (IQR)	1(1,2)	1(1,2)	1(1,2)	1(1,2)	0.061

Values are presented as median (IQR) for continuous variables and number (%) for categorical variables. IQR, inter quartile range; SBP, systolic blood pressure; DBP, diastolic blood pressure; HR, heart rate; eGFR, estimated glomerular filtration rate; TC, total cholesterol; TG, triglyceride; HDL-C, high-density lipoprotein cholesterol; LDL-C, low-density lipoprotein cholesterol; LVEF, left ventricular ejection fraction; ACE, angiotensin-converting enzyme; ARB, angiotensin receptor blocker; CAD, coronary heart disease; STEMI, ST segment elevation myocardial infarction; NSTEMI, non-ST-segment elevation myocardial infarction; UAP, unstable angina pectoris; SAP, stable angina pectoris.

### Serum potassium levels and long-term clinical outcomes

**Figure 2:**
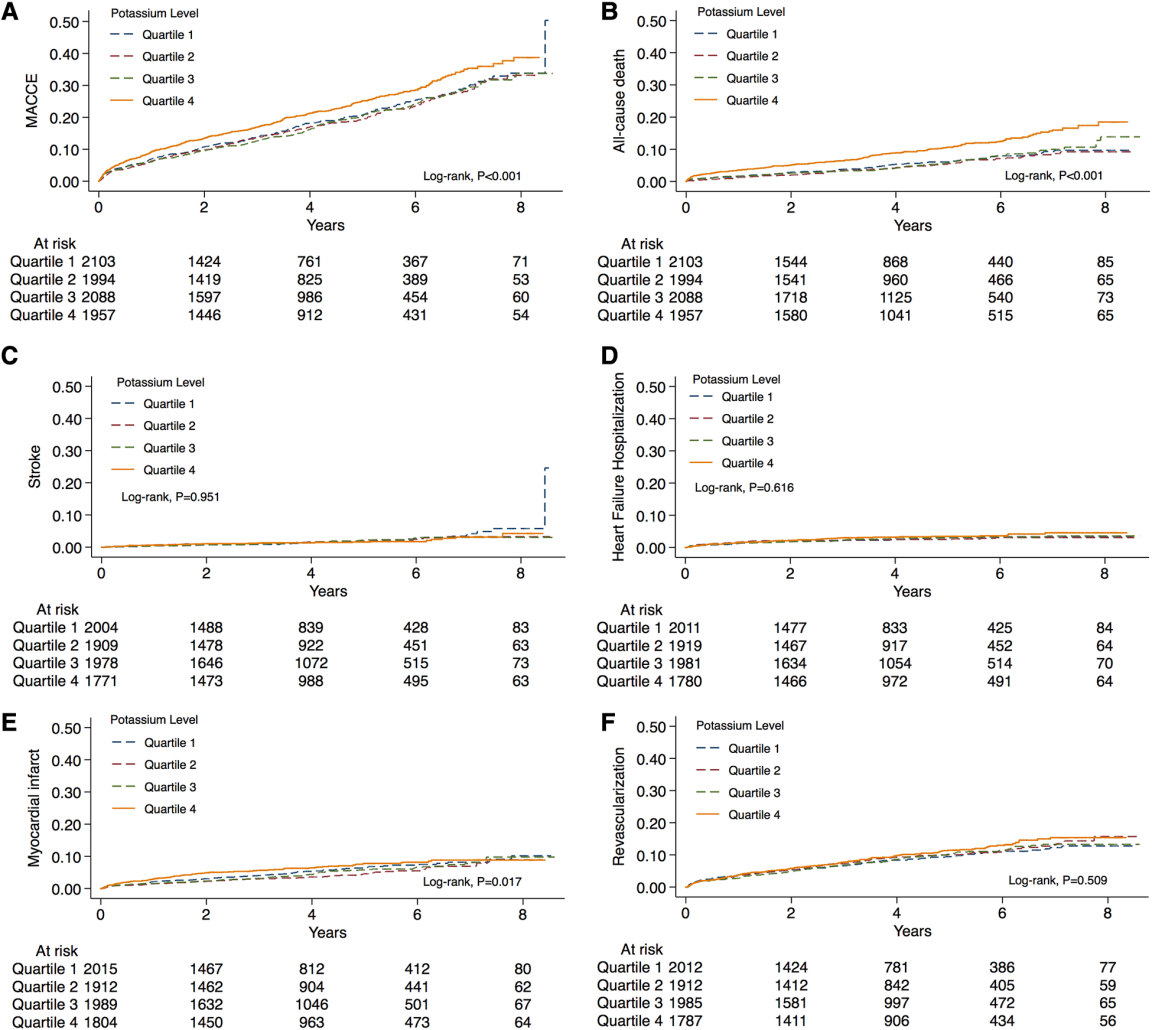
Distribution of the serum potassium levels and Kaplan-Meier curve analysis according to different risk categories. Unadjusted Kaplan-Meier curves during follow-up for (**A**) MACCE, (**B**) all-cause death, (**C**) stroke, (**D**) heart failure hospitalization, (**E**) myocardial infarction, and (**F**) revascularization. MACCE, major adverse cardiovascular and cerebral events.

**Figure 3:**
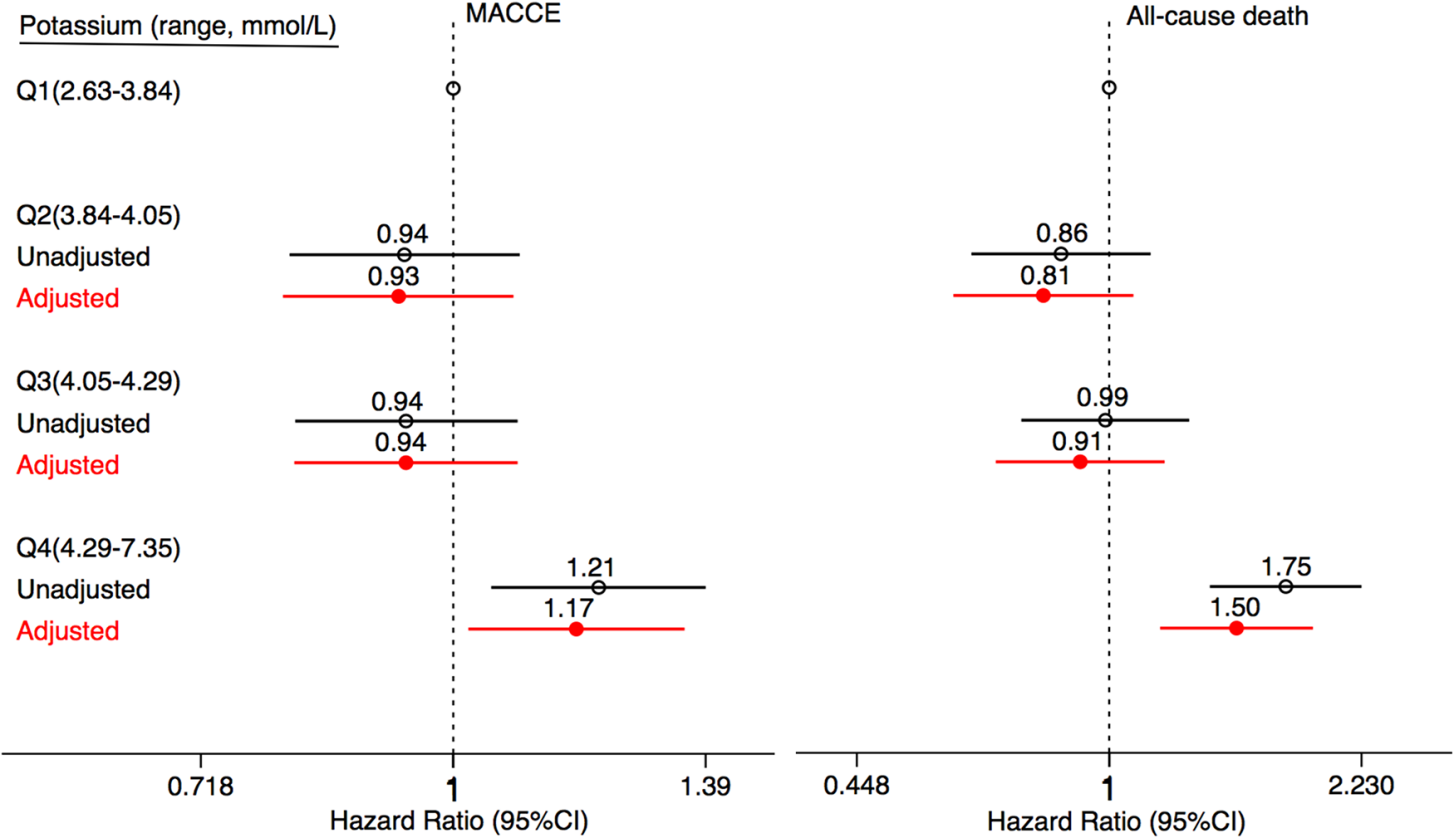
Association between serum potassium levels and clinical outcomes. Forest plots illustrating the HR of MACCE and all-cause death according to the quartiles of serum potassium levels. The HRs are indicated by symbols. The 95% CIs are indicated by line length. HRs were calculated using multivariate Cox regression modeling with adjustments for age, gender, diabetes mellitus, hypertension, prior myocardial infarction and stroke, body mass index, and discharge medications, including aspirin, clopidogrel, ticagrelor, β-blockers, ACE inhibitors/ARB, statins, diuretics, TC, TG, HDL-C, LDL-C, and eGFR. CI, confidence interval; HR, hazard ratio; MACCE, major adverse cardiovascular and cerebral events; ACE, angiotensin-converting enzyme; ARBs, angiotensin receptor blockers; TC, total cholesterol; TG, total triglyceride; HDL-C, high-density lipoprotein cholesterol; LDL-C, low-density lipoprotein cholesterol; eGFR, estimated glomerular filtration rate.

The incidence rate of primary outcomes was 19.7% (*n* = 1,632) during the median follow-up of 4.0 (2.1, 5.8) years. Over the same period, 7.6% (*n* = 631) of patients suffered from all-cause death. Kaplan-Meier estimates of the cumulative incidences of primary outcomes, all-cause death, stroke, MI, HF-related hospitalization, and revascularization are illustrated in Figure 2. Notably, the highest (Q4) quartile of the serum potassium level was associated with a greater risk of MACCE (log-rank, *P < *0.001), all-cause death (log-rank, *P < *0.001), and MI (log-rank, *P = *0.017). The only MACCE component associated with the serum potassium level was total mortality. The difference among quartiles was also nominally statistically significant for MI, but the order of Q4 was different. The results of the Cox Proportional-Hazards model indicated an association of serum potassium level with long-term outcomes (Table 2). Higher serum potassium levels were also associated with both MACCE and all-cause death in unadjusted analyses and even after multivariate adjustment for Model 1 (*P < *0.05 for all). Serum potassium levels exhibited a 20% increased risk of MACCE (HR: 1.20, 95% CI: 1.05–1.38, *P *= 0.008) and a 72% increased risk of all-cause death (HR: 1.72, 95% CI 1.39–2.14, *P *< 0.001), after multivariate adjustment for Model 2. There was no significant trend regarding increased discrimination for either MACCE or all-cause death when serum potassium levels were included in either Model 1 or Model 2 (*P < *0.001 for all). Elevated serum potassium levels were associated with an increased risk of MACCE (4th vs. 1st potassium quartile: HR: 1.21, 95% CI: 1.05–1.39, *P *= 0.008; Figure 3). Following the multivariate Cox model adjustment, an elevated serum potassium level remained independently associated with MACCE (4th vs. 1st potassium quartile: HR: 1.18, 95% CI: 1.02–1.35, *P *= 0.026; Figure 3). In terms of all-cause death, altered serum potassium levels were found to be associated with a higher risk of all-cause death (4th vs. 1st potassium quartile: adjusted HR: 1.50, 95% CI: 1.17–1.91, *P *= 0.001; Figure 3). The results showed that the prognostic difference in potassium levels was confined to comparisons between the lowest (Q1) and highest (Q4) quartiles, whereas the middle quartiles Q2 and Q3 were similar to Q1.

**Table 2:** Cox proportional hazards models for the association of potassium levels and clinical outcomes.

Variable	Model	HR	95%CI	*P*-value	C, base model	*P*-value
MACCE	Unadjusted	1.246	1.086–1.430	0.002	0.520 (0.503–0.537)	
	Model 1	1.184	1.030–1.360	0.017	0.5804 (0.564–0.596)	<0.001
	Model 2	1.201	1.048–1.377	0.008	0.623 (0.608–0.639)	<0.001
All-cause death	Unadjusted	2.085	1.684–2.581	<0.001	0.571 (00.540–0.601)	
	Model 1	1.773	1.421–2.213	<0.001	0.733 (0.709–0.757)	<0.001
	Model 2	1.723	1.388–2.140	<0.001	0.766 (0.743–0.789)	<0.001
Myocardial infarction	Unadjusted	1.265	0.961–1.666	0.094	0.533 (0.499–0.567)	
	Model 1	1.213	0.919–1.600	0.172	0.581 (0.549–0.613)	0.005
	Model 2	1.244	0.951–1.628	0.111	0.666 (0.638–0.693)	<0.001
Heart failure hospitalization	Unadjusted	1.142	0.776–1.682	0.500	0.521 (0.478–0.564)	
	Model 1	1.003	0.679–1.482	0.988	0.627 (0.585–0.670)	<0.001
	Model 2	1.013	0.695–1.477	0.946	0.710 (0.670–0.751)	<0.001
Revascularization	Unadjusted	1.094	0.882–1.357	0.412	0.504 (0.480–0.528)	
	Model 1	1.125	0.906–1.398	0.285	0.523 (0.499–0.546)	0.102
	Model 2	1.144	0.922–1.419	0.221	0.586 (0.563–0.608)	<0.001
Stroke	Unadjusted	0.872	0.528–1.442	0.594	0.491 (0.435–0.548)	
	Model 1	0.799	0.482–1.325	0.385	0.627 (0.576–0.678)	<0.001
	Model 2	0.815	0.491–1.353	0.429	0.691 (0.643–0.740)	<0.001

HR, hazard ratio; CI, confidence interval; MACCE, major adverse cardiovascular and cerebral events; ACE, angiotensin-converting enzyme; ARBs, angiotensin receptor blockers; TC, total cholesterol; TG, total triglyceride; HDL-C, high-density lipoprotein cholesterol; LDL-C, low-density lipoprotein cholesterol; eGFR, estimated glomerular filtration rate.

Model 1: age, gender.

Model 2: Model 1 + diabetes mellitus, hypertension, prior myocardial infarction and stroke, body mass index, and discharge medications, including aspirin, clopidogrel, ticagrelor, β-blockers, ACE inhibitors/ARB, statins, diuretics, TC, TG, HDL-C, LDL-C, and eGFR.

### Serum potassium homeostasis and cardiovascular outcomes

**Figure 4:**
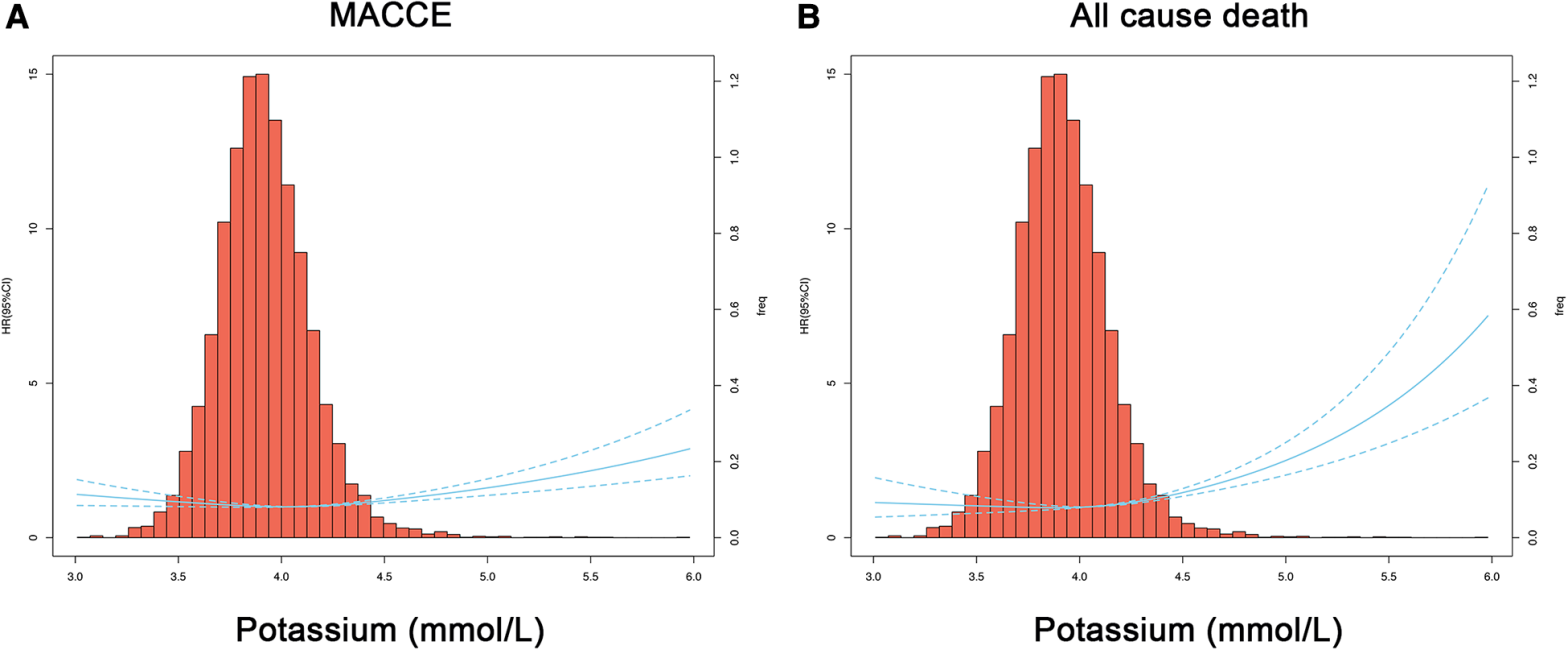
Restricted cubic spline regression models for the serum potassium level and clinical outcome. Hazard ratios and 95% confidence intervals for MACCE (**A**) or all-cause death (**B**) by serum potassium levels, according to restricted cubic spline regression models using 3 knots at 6%, 7%, and 8%. The solid blue line depicts HR, and the dotted blue lines depict 95% CI. The spline curve was superimposed on a histogram showing the frequency of serum potassium levels in the study cohort (right axis). HR, hazard ratio; CI, confidence interval; MACCE, major adverse cardiovascular and cerebral events.

As shown in Figure 4A, we found that the lowest risk of MACCE was linked to an extremely narrow potassium range of 3.99–4.04 mmol/L. When serum potassium levels were higher than 4.00 mmol/L, the risk of MACCE also concomitantly increased, especially at levels ≥4.04 mmol/L. These results suggest that PCI patients with higher serum potassium levels may exhibit poorer outcomes, and at a serum potassium level >5.5 mmol/L the risk of MACCE can increase by 2.17 folds. Furthermore, the risk of all-cause death was the lowest at an optimal serum potassium range of 3.65–4.04 mmol/L (cutoff: 3.87 mmol/L, Figure 4B).

### Serum potassium homeostasis in the ACS subgroup

Our results indicated that the association between serum potassium level and total mortality was confined to patients undergoing PCI for STEMI or unstable angina (Table 3). Supplementary Figure S1 suggested that mortality increases with increasing potassium levels, and not regardless of the serum potassium level. Irrespective of whether patients were suffering from MI or AP, better outcomes were noted in those presenting with a potassium value below 3.83 mmol/L, which may serve as a cut-off value. The risk of all-cause mortality increased significantly with serum potassium levels higher than 3.83 mmol/L. The risk of all-cause death was increased significantly at the serum potassium level higher than 3.83 mmol/L. Therefore, the risk of all-cause death was the lowest in our PCI patients when the serum potassium level was maintained at 3.83 mmol/L (Supplementary Figure S1B). In terms of the occurrence of MACCE (Supplementary Figure S1A), ranges of serum potassium levels were 3.98–4.04 mmol/L in the UAP and 3.77–4.22 mmol/L in the SAP patients. Likewise, regardless of the type of CHD, the serum potassium level of 4.00 mmol/L was used as the cutoff for the MACCE risk stratification.

**Table 3:** Subgroup analysis of Cox proportional hazards models.

	MACCE				All-cause death		
	Model	HR	95%CI	*P*-value	HR	95%CI	*P*-value
STEMI	Unadjusted	1.436	1.154–1.787	0.001	2.397	1.816–3.164	<0.001
	Adjusted	1.296	1.037–1.620	0.023	2.060	1.491–2.847	<0.001
NSTEMI	Unadjusted	1.014	0.745–1.382	0.928	1.224	0.758–1.978	0.408
	Adjusted	1.009	0.752–1.354	0.951	1.054	0.681–1.632	0.814
UAP	Unadjusted	1.186	0.952–1.477	0.128	2.242	1.518–3.312	<0.001
	Adjusted	1.190	0.955–1.483	0.120	1.875	1.260–2.791	0.002
SAP	Unadjusted	1.421	0.752–2.685	0.278	2.319	0.641–8.384	0.200
	Adjusted	1.277	0.673–2.423	0.454	1.703	0.460–6.301	0.426

Model adjusted by age, gender, diabetes mellitus, hypertension, prior myocardial infarction and stroke, body mass index, and discharge medications, including aspirin, clopidogrel, ticagrelor, β-blockers, ACE inhibitors/ARB, statins, diuretics, TC, TG, HDL-C, LDL-C, and eGFR.

HR, hazard ratio; CI, confidence interval; MACCE, major adverse cardiovascular and cerebral events; STEMI, ST segment elevation myocardial infarction, NSTEMI, non-ST-segment elevation myocardial infarction, UAP, unstable angina pectoris; SAP, stable angina pectoris; ACE, angiotensin-converting enzyme; ARBs, angiotensin receptor blockers; TC, total cholesterol; TG, total triglyceride; HDL-C, high-density lipoprotein cholesterol; LDL-C, low-density lipoprotein cholesterol; eGFR, estimated glomerular filtration rate.

## Discussion

In this study, serum potassium levels were associated with higher risks of both MACCE and all-cause death. Notably, we found that higher serum potassium levels increased the risk of MACCE by 1.20-fold and that of all-cause death by 1.72-fold. To the best of our knowledge, this study is the first of its kind that is adequately powered to evaluate associations between the full range of serum potassium levels, MACCE, and all-cause death in PCI subjects, suggesting that maintaining the serum potassium level within the range of 3.8–4.0 mmol/L before PCI might significantly reduce the risk and improve the prognosis of these patients.

Previous studies have linked the serum potassium level with the duration of in-hospital stay (7) and long-term mortality (10, 11) in ACS patients. In the general population, studies have confirmed the association of altered serum potassium levels with increased incidents of adverse cerebrovascular events (12, 13). The most important finding of this study was that the altered serum potassium level might considerably increase the risk of both MACCE and all-cause death in PCI patients. This may in part be attributable to the higher prevalence of conventional cardiovascular risk factors in potassium disorder patients. However, the association between the serum potassium level and risk of MACCE remained significant after adjusting for cardiovascular risk factors, suggesting that the serum potassium level might be an independent risk factor for MACCE and all-cause death. Although the underlying mechanism of dysregulated serum potassium homeostasis, leading to MACCE or even death in PCI patients, has not been comprehensively elucidated, however, it is widely accepted that abnormal serum potassium levels can cause pathological changes in the resting membrane potential of cardiomyocytes, resulting in ventricular arrhythmia (1, 14, 15). It has been hypothesized that the serum potassium level might have a connection with the severity and secondary complications of cardiac patients. Patients with hypertension, HF, or other heart diseases commonly use loop diuretics or thiazide diuretics for a long time, which can lead to hypokalemia (16). Importantly, hyperkalemic patients exhibit significantly higher Cr levels, lower eGFR, and renal insufficiency, which often progress to more serious kidney disease (17). Therefore, the deviances in serum potassium levels may indicate that the patient may have relatively serious systemic diseases and the overall prognosis could be poor. Additionally, these factors can lead to an increased risk of adverse cardiovascular events by a yet unknown mechanism.

From previous reports, it is not clear whether these patients need to be administered potassium supplements before PCI and if so, then what should be the optimal range of serum potassium levels in these patients to lower the risk of MACCE. Some studies suggest that the serum potassium level should be maintained at 4.0–5.0 mmol/L in patients with HF, and potassium supplementation should be provided even if the patient has a low-normal potassium level (serum potassium <4.0 mmol/L) (18, 19). The benefit of a higher level of serum potassium was first observed, though indirectly, during the treatment with Renin-Angiotensis-Aldosterone system (RAAS) inhibitors. What level of serum potassium should be maintained before PCI? In clinical practice, the potassium level is usually maintained at about 4.0 mmol/L. Whether this standard is reasonable and can reduce the occurrence of MACCE needs to be further investigated. Our study indicated a U-shaped relationship between the serum potassium level, risk of MACCE, and all-cause death in PCI patients. Patients who maintained their serum potassium levels within the range of 3.8–4.0 mmol/L exhibited the lowest risk of MACCE and all-cause death. Although we suggest an optimal range of serum potassium levels in PCI, we could not prove a causal relationship between the serum potassium levels and corresponding clinical outcomes. Therefore, whether this study might guide the clinical potassium supplementation strategy in clinical practices needs to be further verified by rigorous randomized controlled trials.

Clinical practice guidelines recommend that the serum potassium level should be either in the range of 4.0–5.0 mmol/L or ≥4.5 mmol/L in acute myocardial infarction (AMI) patients (20). For these patients, the recommended serum potassium level should be maintained at a range of 4.5–5.5 mmol/L. However, a recent study has found that among AMI patients in the intensive care unit, the minimum risk of in-hospital mortality could be associated with a mean potassium level between 3.5 and 4.5 mmol/L (21), which is consistent with the findings of other studies. The meta-analysis of cohort studies has further revealed that both lower (<3.5 mmol/L) and higher (≥4.5 mmol/L) serum potassium levels could be associated with an increased risk of mortality in AMI patients (22). However, it was not clear whether AMI patients undergoing PCI need to maintain a higher serum potassium level (>4.0 mmol/L). Because of the unstable cardiac electrical activities and structural remodeling following an AMI event, malignant arrhythmias such as ventricular tachycardia/ventricular fibrillation are more likely to occur (23). In that context, a higher serum potassium level is more conducive to stabilizing the electrical conductivity in cardiomyocytes. In clinical practice, AMI patients should maintain serum potassium levels above 4.5 mmol/L. Our study indicated that MI could significantly increase the risks of MACCE and all-cause death than AP alone, which is consistent with the current viewpoint. There was a significant association between the serum potassium level and clinical outcomes in MI patients. In this study, regardless of the type of CHD, maintaining the serum potassium level within the range of 3.8–4.0 mmol/L showed significantly lower risks of MACCE and all-cause death, which differs from previous clinical observations.

## Limitations

There are several limitations to this study. First, the analysis of the association between hyperkalemia or hypokalemia and clinical outcomes might have been underpowered because there were fewer participants with hyperkalemia or hypokalemia before PCI. Second, this investigation was a single-center observational study, and confounding factors were relatively difficult to avoid. Third, owing to the inherent limitation of the observational design, residual confounding factors might have influenced the results. Only a single pre-PCI potassium value was available but none during the follow-up. Fourth, this study could not demonstrate a causal relationship between the serum potassium level and the prognosis of PCI patients and whether maintaining the potassium level within the range of 3.8 to 4.0 mmol/L should serve as a therapeutic target for these patients. Finally, our findings were limited to a PCI population and cannot be extended to other populations.

## Conclusions

In summary, our study implies that the highest quartile of serum potassium concentration could be a marker for an adverse prognosis for total mortality in ACS patients undergoing PCI, and the underlying mechanism is currently obscure. In particular, the serum potassium level maintained at 3.8–4.0 mmol/L before PCI might lower the risks of MACCE and all-cause death in these patients.

## Data Availability

The original contributions presented in the study are included in the article/**Supplementary Material**, further inquiries can be directed to the corresponding authors.

## Supplementary material

Supplementary Figure S1 Subgroup analysis of serum potassium levels for predicting MACCE and all-cause death, according to coronary heart diseases. Hazard ratios and 95% confidence intervals for MACCE (**A**) or all-cause death (**B**) by serum potassium levels, according to restricted cubic spline regression models in the subgroups of STEMI, NSTEMI, UAP, and SAP. HR, hazard ratio; CI, confidence interval; MACCE, major adverse cardiovascular and cerebral events; STEMI, ST-elevation myocardial infarction, NSTEMI, non-ST-elevation myocardial infarction, UAP, unstable angina pectoris; SAP, stable angina pectoris.
