# Supplementary figures and images for: Clinical outcomes of serum potassium in patients with percutaneous coronary intervention: insights from a large single-center registry
Source: Front Cardiovasc Med. 2023 Aug 10;10:1216422. doi: 10.3389/fcvm.2023.1216422 (PMC10449252; doi:10.3389/fcvm.2023.1216422)

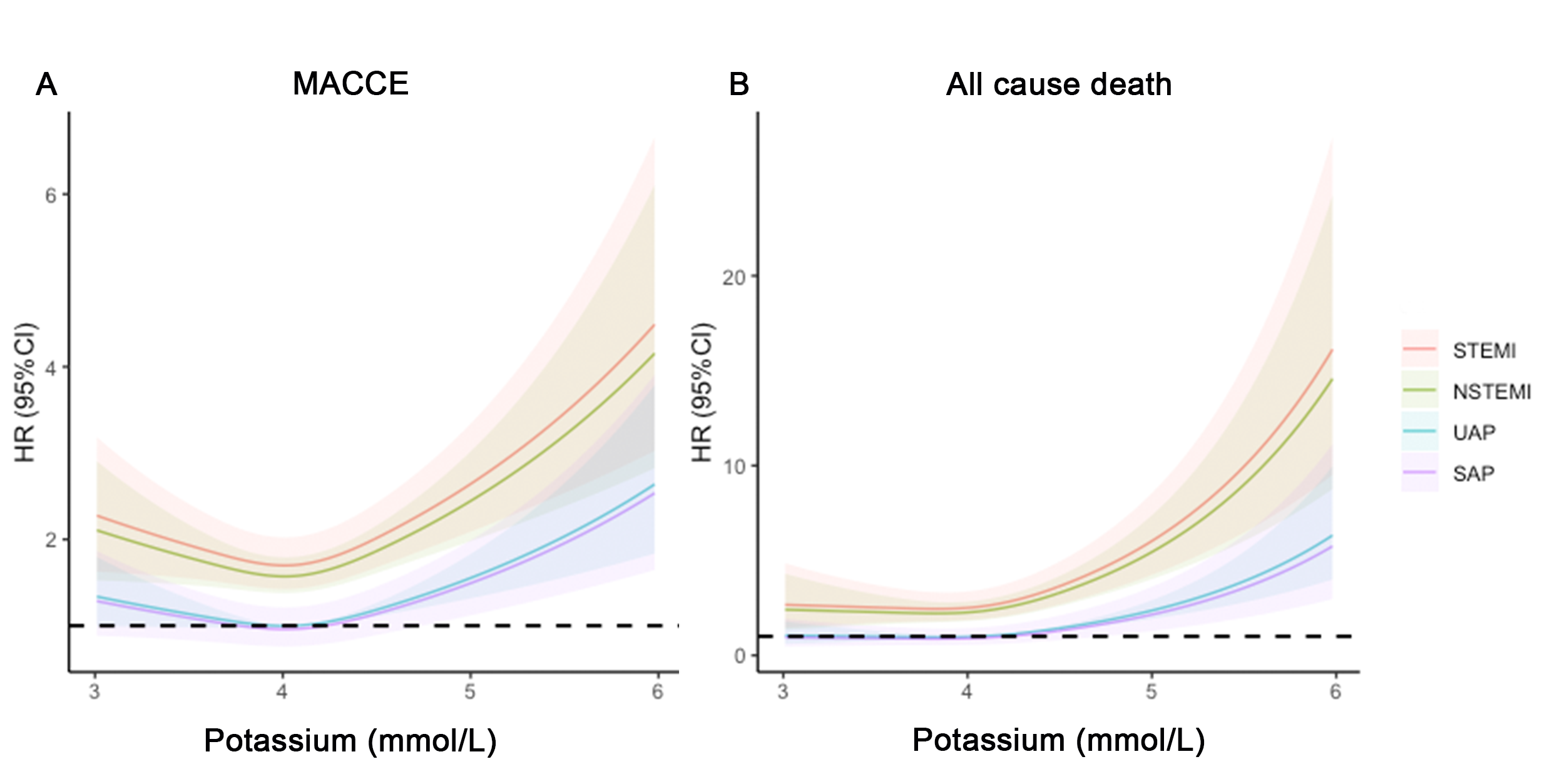

Supplement: Supplementary Figure S1 — Subgroup analysis of serum potassium levels for predicting MACCE and all-cause death, according to coronary heart diseases. Hazard ratios and 95% confidence intervals for MACCE (A) or all-cause death (B) by serum potassium levels, according to restricted cubic spline regression models in the subgroups of STEMI, NSTEMI, UAP, and SAP. HR, hazard ratio; CI, confidence interval; MACCE, major adverse cardiovascular and cerebral events; STEMI, ST-elevation myocardial infarction, NSTEMI, non-ST-elevation myocardial infarction, UAP, unstable angina pectoris; SAP, stable angina pectoris. [file Image1.tif]
